# The ‘Plantain-Optim’ dataset: Agronomic traits of 405 plantains every 15 days from planting to harvest

**DOI:** 10.1016/j.dib.2018.01.065

**Published:** 2018-02-02

**Authors:** Sylvain Dépigny, Frédéric Tchotang, Médard Talla, Désirée Fofack, David Essomé, Jean-Pierre Ebongué, Bernard Kengni, Thierry Lescot

**Affiliations:** a CARBAP, Njombé, Cameroon; b CIRAD, UPR GECO, Njombé, Cameroon; c GECO, Univ Montpellier, CIRAD, Montpellier, France; d CIRAD, UPR GECO, F-34398 Montpellier, France

**Keywords:** Banana, Plantain, Experiment, Agronomic trait, Growth, Development, Variety

## Abstract

The ‘Plantain-Optim’ dataset (came from the ‘Plantain-Optim’ experiment conducted from 2009 to 2011 at CARBAP experimental station in Cameroon. The main objective was to describe agronomic potential of nine plantain varieties, including five natural plantains commonly cropped in Cameroon, and four plantain-like hybrids. A completely randomized bloc permitted to compare growth, development and yield of 45 plants per variety split between five replicates. Cropping practices guarantied non-limiting and homogenous conditions. Each plant was measured every 15 days. Data described aerial organ sizes, foliar structure and bunch characteristics of the mother plant. The ‘Plantain-Optim’ dataset includes the complete individual growth of each studied plantain of the ‘Plantain-Optim’ experiment with a 15-day accuracy. It is a useful standard of plantain varietal diversity for comparison with others datasets. Varietal growth and development homogeneity, biomass production or foliar and bunch structures could be further investigated. Moreover, these accurate data on plantain growth could be valuable for plantain 3D modelling.

**Specification Table**

**Table:** 

Subject area	*Agronomy, Crop modelling, Plant growth and development*
More specific subject area	*Banana, Plantain*
Type of data	*Tables*
How data was acquired	*The ‘Plantain-Optim’ dataset came from the ‘Plantain-Optim’ experiment: data are raw measures from experimental field. Leaf areas were calculated from measured data with the Murray formula* (Murray, 1960) *and the OTO model* (Dépigny, 2015)*.*
Data format	*Raw data*
Experimental factors	*Experimental field was a fallow during more than one year.*
	*Plantlets were choose homogeneous and healthy.*
Experimental features	*The ‘Plantain-Optim’ experiment was a completely randomized bloc with on factor. The factor was the plantain variety with nine modalities. Each variety was represented by 45 plants split into 5 replicates.*
Data source location	*CARBAP experimental station, Njombé, Cameroon*
	*GPS: 4°34'N; 9°38’E; 79* *m a.s.l.*
Data accessibility	*Cirad Dataverse*
	http://dx.doi.org/10.18167/DVN1/CBUVWU

**Value of the data**

• The ‘Plantain-Optim’ dataset describes aerial organ sizes, foliar structure and bunch characteristics of 405 plantains from planting to harvest with a 15-day accuracy.
• The ‘Plantain-Optim’ dataset allows comparing growth and development of nine plantain varieties, included five natural plantains of three different plantain subgroups, and four plantain-like hybrids.
• The ‘Plantain-Optim’ dataset is a useful standard of plantain varietal diversity growth and development for comparison with others plantain datasets.
• The ‘Plantain-Optim’ dataset includes accurate and helpful data to deepen biomass production and allocation, and foliar and bunch structures.
• The ‘Plantain-Optim’ dataset could be basis for plantain 3D modelling.

## 1 Data

### 1.1 Background

Plantain is one of the most important staple food in West and Central Africa. Like others *Musa* species, plantain (*Musa acuminata × Musa balbisiana*) is a giant monocotyledon [1] with an underground corm that supports leaves. Leaf sheaths form the pseudostem. A terminal bud leads the inflorescence, and then the bunch, that is compound by hands of bananas. Bananas are called “fingers”. Farmers commonly cropped a large varietal diversity in West and Central Africa. At this time, varietal explorations led more than 135 plantain varieties, which are preserved in the collection of CARBAP (African Centre for Banana and Plantain Researches) experimental station. This diversity represents a large panel of morphologic traits, which are basis of the existing botanical classification into fours subgroups (French, French horn, False horn and True horn) and three plant sizes (‘small’, ‘medium’ and ‘giant’) [2], [3]. Hypothesis is that this diversity also represents agronomic potential variations. Alongside this botanical knowledge, very few studies explored agronomic differences between plantain varieties, as yield potential, nutrient and water needs or pest and disease sensitivity. Thus, there is a real lack of knowledge and data concerning agronomic potential to benchmark varieties for farmers [4], even more to choose the best suitable varieties for integrating into innovative and sustainable plantain-based cropping systems. The ‘Plantain-Optim’ experiment aimed to produce scientific data to contribute to this basic knowledge.

**Fig. 1:**
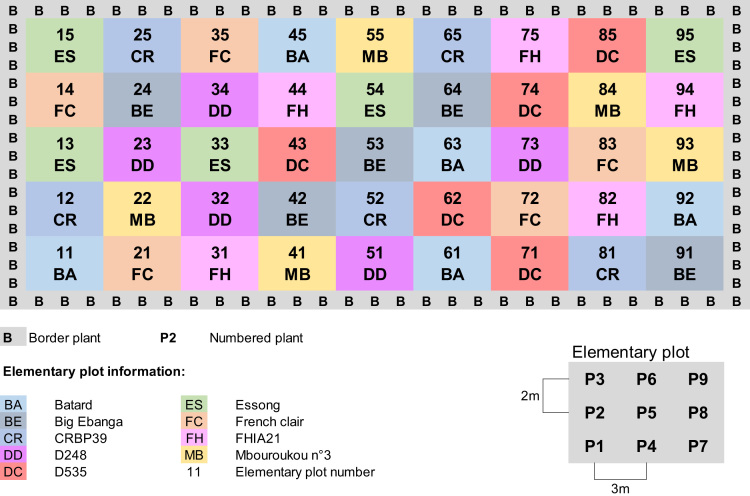
The ‘Plantain-Optim’ experiment design: a completely randomized bloc with a single factor and five replicates. The factor was the variety, with the nine studied varieties as modalities. The experimental plot was compound of 45 numbered elementary experimental plots. Each elementary experimental plot included nine numbered plants of one variety. Each variety was represented by 45 plants.

The ‘Plantain-Optim’ experiment was designed to evaluate and compare yield potential that we assumed to consider as the primary criteria for defining agronomic potential. A board of plantain researchers from CARBAP contributed to identify a panel of commonly cropped and promising varieties in Cameroon, according to future challenges for plantain-based cropping systems. They selected nine varieties, including five natural commonly cropped plantains in Cameroon and four plantain-like hybrids (Table 1). Experimental design was a completely randomized bloc (Fig. 1). The single factor was the variety, with the nine selected varieties as modalities. Five 9-plant replicates led 45 studied plants per variety. A technical guideline allowed nutrient and water inputs, and pest and disease controls, to guaranty non-limiting and homogenous cropping conditions through the 45 elementary experimental plots. Plant growth and development were quantified every 15 days. Aerial organ size assessments included pseudostem height and pseudostem girth every 15 days, and the length and the width of each emitted leaf. Numbers of emitted and living leaves measured every 15 days describe foliar structure dynamic from planting to harvest. Development assessment was based on date recording at each development stages, as planting, different flowering phases, and harvest. Bunch structure assessments included weight, number of hands per bunch, and number of fingers per hand.

**Table 1:** Varieties described in the 'Plantain-Optim' dataset.

Name	Genome	Code	Type	Group	Size
Batard	AAB	BA	Natural	French horn	Giant
Big Ebanga	AAB	BE	Natural	False horn	Medium
CRBP39^a^	AAAB	CR	Hybrid	Hybrid	Medium
D248^a^	AAAB	DD	Hybrid	Hybrid	Medium
D535^a^	AAAB	DC	Hybrid	Hybrid	Medium
Essong	AAB	ES	Natural	French	Giant
French Clair	AAB	FC	Natural	French	Medium
FHIA21^b^	AAAB	FH	Hybrid	Hybrid	Medium
Mbouroukou no. 3	AAB	MB	Natural	False horn	Medium

a Plantain-like hybrid from CIRAD-CARBAP breeding collaborative program.

b Plantain-like hybrid from FHIA breeding program.

These data were used to model each plantain variety growth and development in order to better understand factors that affected yield. This analysis highlighted that photosynthetic efficiency of natural plantains was higher than for plantain-like hybrids [4]. It was a first step in understanding agronomic differences among plantain varietal diversity. Another current analysis aims to describe variations of agronomic traits during crop cycle among these varieties. The dataset sharpness, as the sizes of 15,000 leaves, also permitted to deeply explore plantain leaf area dynamic and develop the OTO model [5]: it is a useful tool for estimating banana leaf area with the only sizes of three leaves whatever variety, development stage and cropping conditions. In addition, according to plantain dataset scarcity, we think the ‘Plantain-Optim’ dataset could be a useful reference for others studies to compare growth and development of the studied varieties according to environment and cropping conditions. Furthermore, we envision that these data could be used to deepen varietal growth and development homogeneity, biomass production and allocation, or foliar and bunch structures among plantain diversity. The ‘Plantain-Optim’ dataset contains also basic information for plantain 3D modelling, which could be helpful for better understanding plant functioning or interactions between plantains and associated crops into multi-species cropping systems.

### 1.2 Dataset description

The ‘Plantain-Optim’ dataset languages are English and French. It is available through Dataverse Cirad (http://dataverse.cirad.fr) under the CC0 waiver. Data can be downloaded through six tab-delimited txt files. Data caption for each file can also be download in English and French language; six caption files are available for each language.

Three files describe mother-plant growth, flowering and harvest, respectively ‘optim_mp-growth.txt’, ‘optim_mp-flowering.txt’ and ‘optim_mp-harvest.txt’. Each file begins with a complete plant description in the first six columns:

– Column 1: experimental number of the plant (Variable: Plant). It ranges among 405 three-digit values defined by the number of the elementary experimental plot concatenated with the number of the plant into this elementary experimental plot;
– Column 2: number of the elementary experimental plot of the plant (Variable: Plot). It ranges among 45 two-digit values defined by the row and column position into the experimental plot (Fig. 1);
– Column 3: plant variety (Variable: Var). It ranges among 9 two-character codes presented in Table 1;
– Column 4: plant variety type (Variable: Type). It ranges among 2 three-character types presented in Table 1;
– Column 5: plantain subgroup of the plant (Variable: Group). It ranges among 4 three-character groups presented in Table 1;
– Column 6: plant position into the elementary experimental plot (Variable: Pos). It ranges from 1 to 9 (Fig. 1).

**Fig. 2:**
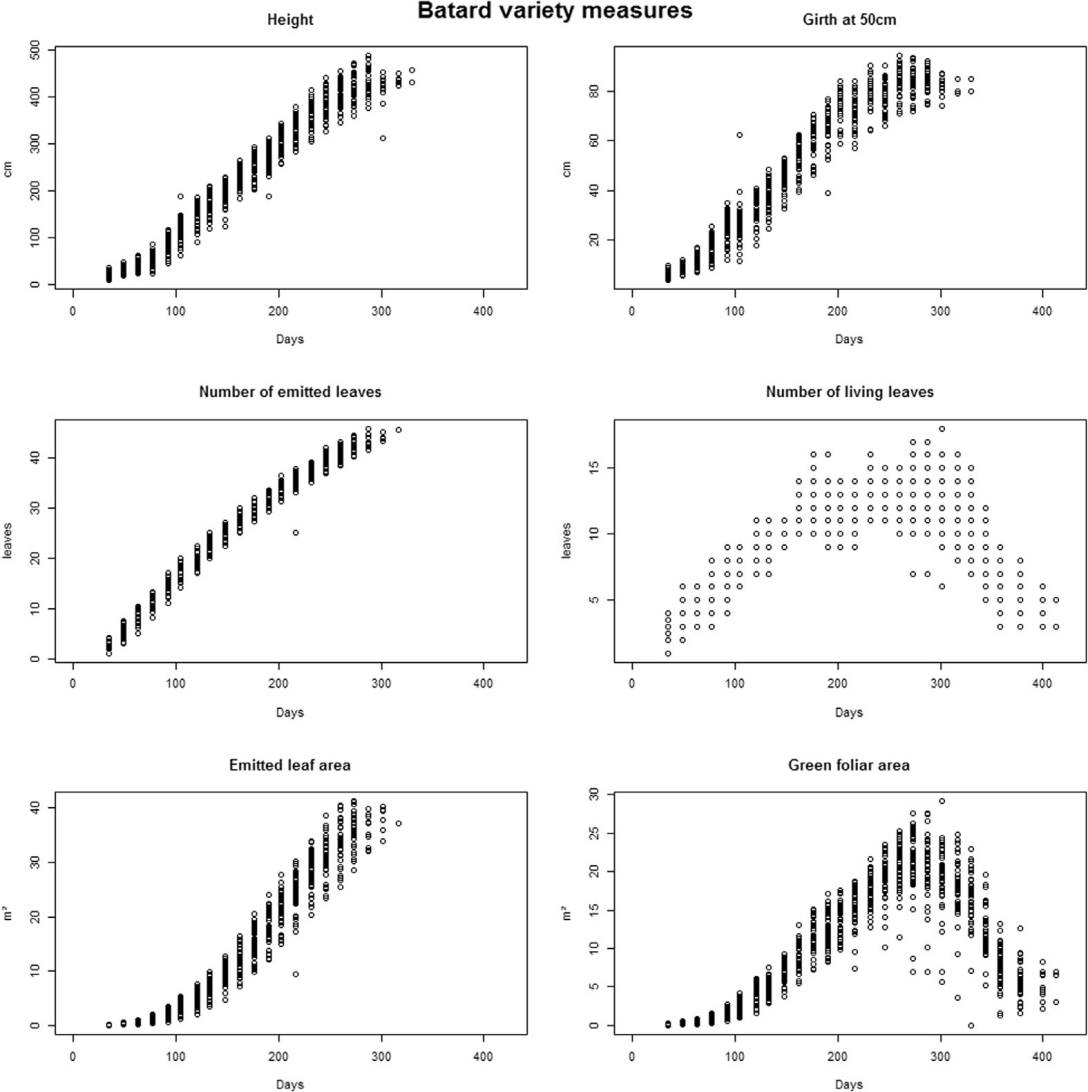
Raw values of six agronomic traits over the time for the variety Batard. A dot represents a dated value of one of the 45 plants of the variety Batard for the focus agronomic trait. Height, girth at 50 cm above soil, number of emitted leaves and number of living leaves were measured on the experimental field. Emitted leaves area and green foliar area were calculated from leaf sizes and numbers with the Murray formula and the OTO model.

The file ‘optim_mp-growth.txt’ contains growth data. It is a table with 13,366 rows and 13 columns. The first row contains headers. Every 15 days, each plant is described by pseudostem height in cm (Variable: Ht), pseudostem girth at 50 cm above soil in cm (Variable: G50), number of emitted leaves (Variable: NEL), emitted leaf area (Variable: ELA), number of living leaves (Variable: NLL) and green foliar area in m^2^ (Variable: GFA). Fig. 2 shows as example agronomic trait values of each plant over the time for the variety Batard. The variable ‘DAP’ (Days After Planting) represents time: from planting to the last harvested plant, data are split between 33 observation dates.

**Fig. 3:**
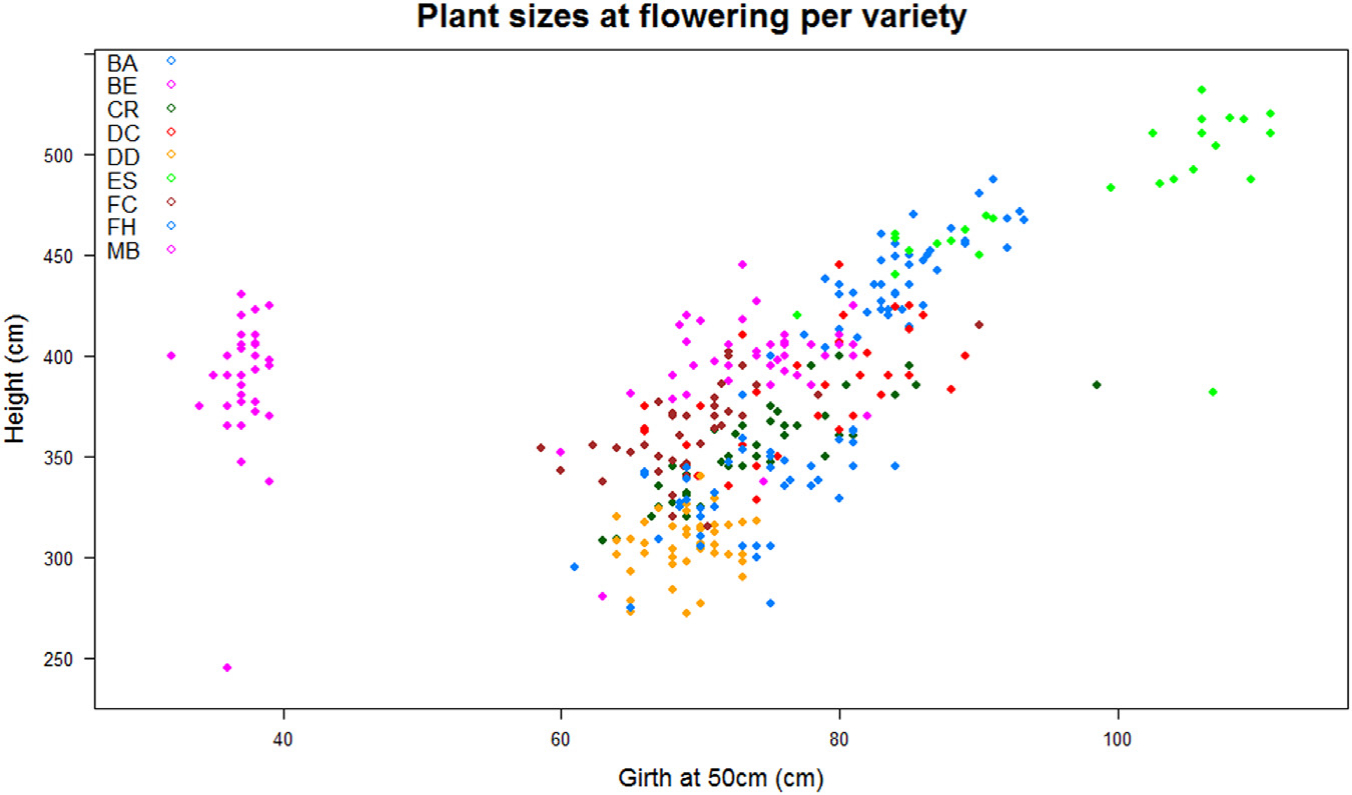
All observed plant size at flowering, in particular height and girth at 50 cm above soil. Each dot represents one plant of the ‘Plantain-Optim’ experiment. Colours represented the nine different studied varieties.

The file ‘optim_mp-flowering.txt’ contains data that describe plants at flowering. It is a table with 406 rows and 15 columns. The first row contains headers. Four variables describe development periods: planting-flowering period in days (Variable: PFP), flower pointing-flowering period in days (Variable: F1FP), flower pointing-horse head period in days (Variable: F1F2P) and horse head-flowering period in days (Variable: F2FP). Four variables present agronomic traits of plants at flowering: pseudostem height in cm (Variable: Htf), pseudostem girth at 50 cm above soil in cm (Variable: G50f), number of emitted leaves (Variable: NELf), number of living leaves (NLLf) and total leaf area emitted from planting in m² (Variable: TEFAf). Fig. 3 shows as example all observed plant size at flowering per variety. Missing values are recorded ‘NA’.

**Fig. 4:**
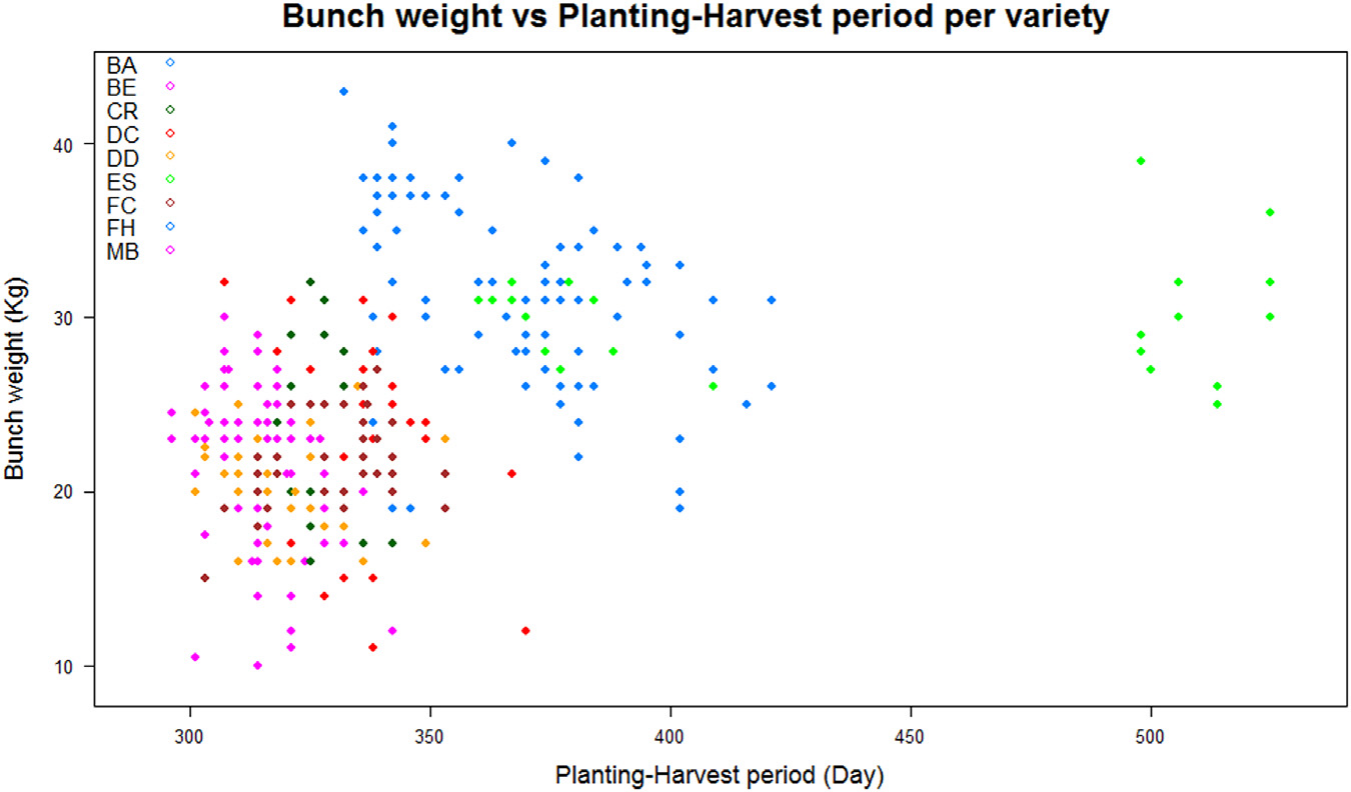
All bunch harvested and weighted from the ‘Plantain-Optim’ experiment are dotted according to their planting-harvest period. Each dot represents one plant of the ‘Plantain-Optim’ experiment. Colours represented the nine different studied varieties.

The file ‘optim_mp-harvest.txt’ contains data that describe plants at harvest. It is a table with 406 rows and 27 columns. The first row contains headers. Four variables describe development periods: flowering-harvest period in days (Variable: FHP1), flowering-harvest period in degrees (Variable: FHP2), planting-harvest period in days (Variable: PHP1) and planting-harvest period in degrees (Variable: PHP2). One variable leads number of living leaves at harvest (Variable: NLLh). Sixteen variables describe each bunch: bunch weight in kg (Variable: BW), number of hands of the bunch (Variable: NHB), number of fingers of the bunch (Variable: NFB) and thirteen variables that lead the number of fingers per hand from the top to the bottom of the bunch (Variables: NFH1 to NFH13). Fig. 4 plots as example bunch weight versus planting-harvest period for all observed plants per variety. Missing values are recorded ‘NA’.

The file ‘optim_sucker-growth.txt’ describes sucker growth from its selection to mother-plant harvest. It is a table with 11,746 rows and 11 columns. The first row contains headers. It also begins with the first six columns that describe each plant. Every 15 days, each sucker is described by pseudostem height in cm (Variable: Hts), pseudostem girth at 50 cm above soil in cm (Variable: G50s), number of emitted leaves (Variable: NELs) and number of living leaves (Variable: NLLs). The variable ‘DAP’ (Days After Planting) represents time: from the first sucker selected in the experiment to the last harvested mother-plant, data are split between 29 observation dates. Missing values are recorded ‘NA’.

Two files contain leaf data of mother-plants. The file ‘optim_mp-leaves-length.txt’ contains length in cm of each leaf of each 405 plants of the ‘Plantain-Optim’ experiment. It is a table with 406 rows and 55 columns. The first row is the leaf rank from the bottom to the top of the plant. The first column is the 3-digit plant number in the ‘Plantain-Optim’ experiment. The file ‘optim_mp-leaves-width.txt’ has exactly the same structure but contains width in cm of each leaf of each 405 plants of the ‘Plantain-Optim’ experiment. Missing values are recorded ‘NA’.

## 2 Methods

### 2.1 Varietal panel definition

The first objective was to identify a panel of representative plantain varieties. To be studied a variety had to be either commonly cropped in Cameroon or promising with interesting characteristics as bunch structure or tolerance for a disease. A board of plantain researchers from CARBAP, compounded by four agronomists, one entomologist, two phytopathologists, two nematologists, one breeder, two socioeconomists and one post-harvest researcher, contributed to select the studied varieties. Each researcher choose among plantain varietal diversity ten interesting and promising varieties according to his own scientific expertise. All propositions were ranked to lead the final list of nine varieties, that included five natural plantains commonly cropped in Cameroon and four plantain-like hybrids (Table 1). We assumed that True horn type subgroup was excluded because of its small number of fingers per bunch. However, the nine selected varieties represent the three most cropped subgroups into plantain varietal diversity.

### 2.2 Experiment design and cropping

The second objective was to compare yield potential between selected varieties. The same environmental and cropping conditions had to be applied to each studied variety. The CARBAP experimental station in Cameroon provided a homogeneous experimental plot of 3000 m^2^, which was a fallow for more than one year. Soil is a brown andosol derived from a volcanic platform. Climate is humid-topical with an 8-month rainy season from mid-March to mid-November and a 4-month dry season. Soil and climate conditions were assumed homogeneous within experimental plot. Thus, experimental design could be a completely randomized bloc. The single factor was the plantain variety. Modalities were the nine selected varieties. Each elementary plot included nine numbered plants of the same variety, three 3-lines of plants arranged in a 2 × 3 m^2^ pattern. Five replicates represented each studied variety, and thus led 45 plants per variety. Number of plants per elementary plot and number of replicates were chosen according to recommendation for experimentation with bananas [6]. This size aimed to ensure a sufficient number of studied plants, including all growing accident. The experiment was conducted from June 2009 to December 2011. However, the ‘Plantain-Optim’ dataset only concerns data assessed from planting (July 2009, 29th) to harvest of the first crop cycle of each variety; the last mother-plants were harvested on February 2011. Thus, it mainly concerns mother-plant data.

Plant materials were healthy and homogeneous plantlets that were produced by a pathogen-free in vivo vegetative method [7]. Non-limiting cropping conditions were achieved by providing systematically mineral nutrition, irrigation, and control of weeds, pests and diseases. Mineral nutrition was calculated from local recommendations for sweet banana monoculture and adapted to provide quantities of nutrients in excess of known plantain needs [8]. Fertilization followed suggested distribution [9] during crop cycle and was applied twice a month to limit nutrient leaching. Each plant received an average of 270 g nitrogen, 140 g phosphorus, 900 g potassium, 400 g calcium, 300 g magnesium and 100 g sulphur per year. Irrigation was applied every two days during the complete dry season. Weeds were eliminated by manual clearing and herbicide applications from the third month after planting. Black Sigatoka disease (*Mycosphaerella fijiensis*) was controlled by manual pruning of diseased leaves and weekly fungicide application. Plant-parasitic nematodes (*Radopholus similis* in particular) were controlled by nematicide application every 3 months. Banana weevils (*Cosmopolites sordidus*) were controlled by high-density pheromone traps, which were weekly emptied, and insecticide applications. Accurate plant practices, as early sucker selection, 15-days desuckering or wiring, led to maintain a homogeneous plantain population. None significant pest and disease attack was registered during data collection. Leaf analysis results at flowering of a sample of leaves of studied varieties showed high values and confirmed nutrition stress absence.

### 2.3 Data assessment

Each plant was identified by a 3-digits experiment number (Variable: Plant). The two first digits represent the number of its elementary experimental plot (Variable: Plot). The third digit represents the plant number in the elementary experimental plot from one to nine (Fig. 1). Each plant was also characterized by its varietal code (Variable: Var), varietal type (Variable: Type), plantain subgroup (Variable: Group) and position in the elementary experimental plot (Variable: Pos). A plant was compounded by the mother-plant (planted plant) and its selected sucker (second cycle plant from layering). The dataset ‘Plantain-Optim’ mainly concerns the mother-plant, but also integrates sucker information until the mother-plant harvest.

#### 2.3.1 Development stages recording

Planting, flowering and harvest dates were recorded. Flowering was split between three stages: i) “flower pointing” (F1), i.e. the day when the terminal bud appeared between the two last emitted leaves, ii) “horse head” (F2), i.e. the day when the flower toggled, and “complete flowering” or “flowering” (F), i.e. the day when the latest fertile hand was appeared. Harvest was decided when at least one finger of the first hand had turned into yellow. These dates allowed calculating the planting-flowering period (Variable: PFP), i.e. number of days between planting and flowering, the flowering-harvest period (Variable: FHP1), i.e. number of days between flowering and harvest, and the planting-harvest period (Variable: PHP1), i.e. number of days between planting and harvest. Number of days between different flowering stages (Variables: F1F2P; F2FP; F1FP) were also calculated by the same method. The planting-harvest period (Variable: PHP2) and the flowering-harvest period (Variable: FHP2) were also converted into thermic sum by adding daily temperatures greater than 0 °C.

#### 2.3.2 Growth measurements

Each mother-plant was measured twice every month from planting to harvest, totalling 24 to 35 measures per plant per cycle according to variety. Each observation concerned height and girth of pseudostem, number of emitted leaves and number of living leaves. Pseudostem height (Variable: Ht) was measured between the soil and the bottom of the “V” formed by the two last emitted leaves. Pseudostem girth (Variable: G50) was measured at 50 cm above soil. Each new leaf had its rank burned on sheath. Number of emitted leaves (Variable: NEL) was the rank of the latest emitted leaf. Number of living leaves (Variable: NLL) was the difference between the youngest leaf rank and oldest leaf rank. The first leaf was the first emitted leaf with width superior to 10 cm. The latest emitted leaf occurred before the first floral bract also call “false leaf”. A leaf was counted living if it had more than 50% of green area. Each emitted leaf had its length and width measured. Leaf length was measured along the main midrib from the base to the tip of blade. Leaf width was assessed at the widest part of its blade. For each emitted leaf, length and width were used to calculated the leaf area with the Murray formula [10]. At each observation date, leaf area sum of emitted leaves led the emitted leaf area (Variable: ELA) and leaf area sum of living leaves led the green foliar area (Variable: GFA). For selected sucker, pseudostem height (Variable: Hts), pseudostem girth (Variable: G50s), number of emitted leaves (Variable: NELs) and number of living leaves (Variable: NLLs) were recorded with the same methodology twice every month from its appearance to mother-plant harvest.

#### 2.3.3 Bunch data

Each harvested bunch was weighted (Variable: BW) in the field with an electronic scale with an accuracy of one tenth of kilogramme. Number of hands per bunch (Variable: NHB) and total number of fingers per bunch (Variable: NFH) were counted and recorded. Number of fingers of each hand (Variables: NFH1 to NFH13) of the bunch was also counted and recorded.

A strong data process was applied to minimize data errors. Controls twice a month permitted to verify and adjust if necessary applied methods during measures into experimental field. The first control on measurement report occurred the same day than measurements: the observation team verified all data of the day at office to spot and correct outliers. The second control occurred when data entering by the computer team: each outlier that was found was validated with the observation team. The third verification was a random comparison between measurement reports and electronic data; more than five errors by variable for the 405 studied plants (error ≤ 1.2%) engaged a total verification by both observation and computer teams. The last verification involved descriptive statistical analysis: outliers were systematically checked and compare with measurement reports. Maxima, minima, means and variances were calculated and checked for all variables. Important variation engages a verification of the total variable values with measurement reports. Variables were also plotted to spot outliers. During all the verification process, outliers without correction from measurement reports were deleted.
