# Supplementary material for: The ‘Plantain-Optim’ dataset: Agronomic traits of 405 plantains every 15 days from planting to harvest
Source: Data Brief. 2018 Feb 2;17:671–80. doi: 10.1016/j.dib.2018.01.065 (PMC5852287; doi:10.1016/j.dib.2018.01.065)
Supplement: Supplementary file 1 — Supplementary material [file mmc1.docx]

**Conflict of interest**

The authors declare no competing financial interest.
